# *Toxoplasma* infection and Rhesus blood group system: A systematic review and meta-analysis

**DOI:** 10.1371/journal.pone.0287992

**Published:** 2023-07-05

**Authors:** Tooran Nayeri, Mahmood Moosazadeh, Abdolhossein Dalimi Asl, Fatemeh Ghaffarifar, Shahabeddin Sarvi, Ahmad Daryani

**Affiliations:** 1 Toxoplasmosis Research Center, Communicable Diseases Institute, Mazandaran University of Medical Sciences, Sari, Iran; 2 Department of Parasitology, School of Medicine, Mazandaran University of Medical Sciences, Sari, Iran; 3 Gastrointestitional Cancer Research Center, Non-communicable Disease Institute, Mazandaran University of Medical Sciences, Sari, Iran; 4 Department of Parasitology and Entomology, Faculty of Medical Sciences, Tarbiat Modares University, Tehran, Iran; Mashhad University of Medical Sciences, ISLAMIC REPUBLIC OF IRAN

## Abstract

**Background:**

Toxoplasmosis is one of the most common infections in humans and animals, which is caused by an obligate intracellular opportunistic parasite known as *Toxoplasma gondii* (*T*. *gondii*). Some data have shown that both Rhesus (Rh)-positive and Rh-negative individuals differ in response to biological factors, including *Toxoplasma* infection. Therefore, this systematic review and meta-analysis was conducted to investigate the scientific evidence regarding the possible association between the Rh blood group and *Toxoplasma* infection and to determine the seroprevalence of *T*. *gondii* in the Rh blood group system.

**Methods:**

The research was conducted on PubMed, ScienceDirect, ProQuest, and Google Scholar databases until January 2023. Twenty-one cross-sectional studies were included with a total of 10910 people. The data were synthesized using a random effect model with 95% confidence intervals (CIs).

**Results:**

The overall prevalence of *T*. *gondii* was calculated at 32.34% (CI 95%: 28.23–36.45%) and 33.35% (CI 95%: 19.73–46.96%) in Rh-positive and Rh-negative blood groups. In addition, the pooled OR for the relationship between the Rh blood group and the seroprevalence of *T*. *gondii* was 0.96 (95% CI: 0.72–1.28).

**Conclusions:**

This meta-analysis showed a high prevalence of *Toxoplasma* infection in both Rh-negative and positive blood groups. This systematic review and meta-analysis revealed that no significant association was found between toxoplasmosis and Rh factor. Because of the limited number of studies in this field, more research is recommended to determine the exact relationship between toxoplasmosis and the Rh factor.

## Introduction

Toxoplasmosis is a global parasitic disease caused by a single-celled obligate coccidian parasite in the phylum Apicomplexa [1]. Humans and any warm-blooded animal can be infected with *Toxoplasma gondii* (*T*. *gondii*), and the prevalence of this infection in different countries varies between 5 and 80% depending on weather conditions, health standards, and feeding habits [2]. This parasite has been reported even in cold-blooded animals [3]. The *Toxoplasma* infection is typically a chronic disease with no clinical symptoms or with self-limiting symptoms in immunocompetent persons. However, in congenital disorders, the infection can cause microcephaly, hydrocephalus, mental retardation, cerebral calcification in the developing fetus, stillbirth, abortion, and fetal death [4]. Additionally, immunosuppressed patients may experience serious clinical complications, such as encephalitis, pneumonia, and disseminated systematic disease [5, 6]. Toxoplasmosis also may lead to psychotic symptoms and changes in the personality of individuals [7–11]. The main routes of transmission of *Toxoplasma* infection include consumption of food or water contaminated with oocysts shed by cats, ingestion of tissue cysts of raw or undercooked meat, and through the placenta during pregnancy [12, 13]. Organ transplants or injections of infected blood products are also other ways to transmit the infection [14, 15]. This organism is capable of surviving up to 50 days in citrated whole blood stored at 4°C [16]. Human blood is categorized into two primary systems [ABO blood type and the Rhesus (Rh) blood group] [17]. The Rh blood type is the second most important blood group (next to the ABO system). The Rh system has 44 or more distinct antigens, but the presence or absence of the D antigen in red blood cells is clinically the major cause of polymorphism [18]. Antigen D is absent in approximately 16% of the population (Rh-negatives) because of the removal or replacement of antigen D [19]. According to structural homology data, protein D is an ion pump whose specificity and physiologic function are unknown [20, 21]. Several studies have shown that the relation between *Toxoplasma* infection and human response time, personality, and physiology is influenced by the Rh phenotype in the infected person [22–26]. One study also showed that Rh-negative *Toxoplasma*-infected people are about three times more likely to have a traffic accident than Rh-negative *Toxoplasma*-free people or Rh-positive people [25]. Therefore, the main goal of this systematic review and meta-analysis was to determine the prevalence of *T*. *gondii* in the Rh blood group and to investigate the relationship between seroprevalence of anti-*T*. *gondii* antibodies and the Rh factor.

## Methods

### Design and protocol registration

The review was performed in accordance with Preferred Reporting Items for Systematic Reviews and Meta-analyses guidelines (PRISMA) (S1 Checklist) [27]. The details of the protocol have been registered on the website of the International Prospective Register of Systematic Reviews with the registration number CRD42023390995.

### Information sources and search strategy

Four English language databases of the following websites: (www.pubmed.gov), (www.sciencedirect.com), (www.search.proquest.com), and (www.scholar.google.com) were searched from inception until January 2023. The search process was accomplished based on the medical subject heading terms: “*Toxoplasma gondii*”, “toxoplasmosis”, “blood donors”, “blood group”, “Rhesus”, and “RhD” (S1 File). A manual search of references from recent reviews and relevant published original studies was done to find more relevant articles. The EndNote file (EndNote X9, Thomas Reuters, Philadelphia, PA, USA) was used for managing the references. No geographical or time restrictions were applied. However, language restriction was applied and only articles in the English language were included in the study.

### Inclusion and exclusion criteria

The inclusion criteria included: (1) studies published until January 2023, (2) cross-sectional studies that reported the prevalence of *Toxoplasma* infection in the Rh blood group with various diagnostic methods, (3) original research papers, and (4) studies with available full texts in the English language. The exclusion criteria were: (1) studies with no exact information about the sample size, (2) the review, systematic review, and meta-analysis articles, (3) gray literatures, (4) non-human studies, (5) dissertations, and (6) conference papers.

### Study selection

Two independent authors carefully examined titles and abstracts. The full text of the papers identified considered as relevant based on the title and abstract were independently reviewed by the same two reviewers. The third author resolved discrepancies between the reviewers.

### Quality assessment

The quality of the included studies was evaluated using the Newcastle-Ottawa scale (NOS) [28]. Each report was rated as low (scores of < 3), moderate (scores of 3–5), and high (scores of 6–7) quality.

### Data extraction

The following information was extracted from each study: the first author, publication year, place of study, diagnostic methods, type of antibody, sample size, number of people with Rh-positive blood group, number of people with Rh-negative blood group, number and percentage of people with Rh-positive blood group and *Toxoplasma* positive, and number and percentage of people with Rh-negative blood group and *Toxoplasma* positive.

### Statistical analysis

This meta-analysis was done with Stata software (version 14; Stata Corp, College Station, Texas, USA). The prevalence of *Toxoplasma* infection was calculated based on data from each study. The risk of *Toxoplasma* infection in the Rh blood group was estimated by odds ratio (OR). It was considered statistically significant when P < 0.05. The I^2^ statistic was used to evaluate the heterogeneity and I^2^ > 50% was considered as high heterogeneity [29]. If there is no heterogeneity in the literature, the fixed effects model is employed, and otherwise, the random effects model is used. Egger’s regression test and funnel plot were applied to display the publication bias and small study effects. Sensitivity analysis was carried out by altering the inclusion criteria in this meta-analysis. In addition, analysis of the subgroups was conducted according to the diagnostic methods, place of study, and NOS score.

## Results

### Study identification and selection

**Fig 1:**
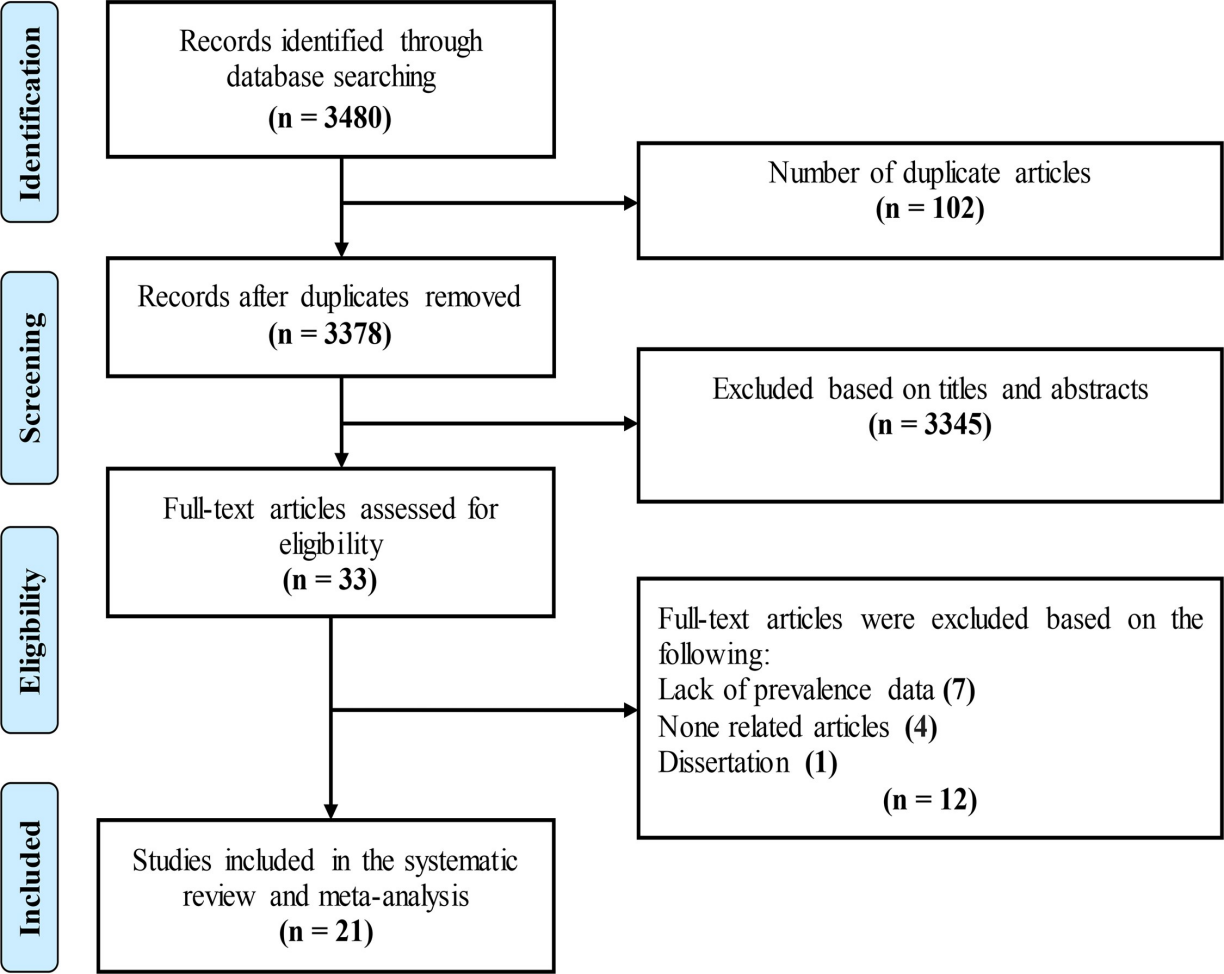
The PRISMA flow diagram describing the study design process.

As shown in Fig 1, 3480 relevant studies were obtained from the literature search, and after removing duplicates and initial screening of titles and abstracts, 33 studies remained. After a complete review of the text, 1 thesis and 11 articles were excluded due to the lack of prevalence data or irrelevant data. Eventually, a total of 21 eligible studies were included in this systematic review and meta-analysis with respect to the inclusion and exclusion criteria. Characteristics of the included publications are listed in Table 1.

**Table 1:** Baseline characteristics of the included cross-sectional studies in this systematic review and meta-analysis.

Id	First author (Publication year)	Place of study	Diagnostic methods	Type of antibodies	Sample size (n)	Rh^+^ (n)	Rh^+^ & *Toxoplasma*+ (n, %)	Rh^-^ (n)	Rh^-^ & *Toxoplasma+* (n, %)
1	Flegr *et al*. (2008) [23]	Czech Republic	ELISA	IgG and IgM	336	270	45 (16.66)	66	13 (19.69)
2	Novotná *et al*. (2008) [22]	Czech Republic	CFT and ELISA	IgG and IgM	868	668	224 (33.53)	200	63 (31.5)
3	Flegr *et al*. (2009) [25]	Czech Republic	CFT and ELISA	IgG and IgM	3890	3169	709 (22.37)	721	181 (25.1)
4	Flegr *et al*. (2010) [24]	Czech Republic	CFT and ELISA	IgG and IgM	292	220	71 (32.27)	72	22 (30.55)
5	Flegr *et al*. (2013) [30]	Czech Republic	CFT and ELISA	IgG and IgM	490	405	125 (30.86)	85	28 (32.94)
6	Obaid *et al*. (2014) [31]	Iraq	ELISA	IgG and IgM	102	79	27 (34.17)	23	3 (13.04)
7	Sarkari *et al*. (2014) [32]	Iran	EIA	IgG and IgM	1469	1354	263 (19.42)	115	21 (18.26)
8	Jafari Modrek *et al*. (2014) [33]	Iran	ELISA	IgG and IgM	375	285	73 (25.61)	90	21 (23.33)
9	Siransy *et al*. (2016) [34]	Ivory Coast	ELISA	IgG	106	94	64 (68.08)	12	4 (33.33)
10	Obaid *et al*. (2017) [35]	Iraq	ELISA	IgG and IgM	91	69	16 (23.18)	22	1 (4.54)
11	Abd El Wahab *et al*. (2018) [36]	Egypt	ELISA	IgG and IgM	276	250	138 (55.2)	26	12 (46.15)
12	Sadik Smael *et al*. (2018) [19]	Iraq	LAT and ELISA	IgM	200	133	2 (1.5)	67	65 (97.01)
13	Flegr *et al*. (2018) [37]	Czech Republic	CFT and ELISA	IgG and IgM	123	76	38 (50)	47	24 (51.06)
14	Manouchehri Naeini *et al*. (2019) [38]	Iran	ELISA	IgG	385	258	97 (37.59)	127	49 (38.58)
15	Henin (2019) [39]	Egypt	ELISA	IgG and IgM	200	178	120 (67.41)	22	10 (45.45)
16	Lachkhem *et al*. (2020) [40]	Tunisia	IFA and ELISA	IgG and IgM	800	725	321 (44.27)	75	34 (45.33)
17	Noori Al-Tufaili *et al*. (2020) [41]	Iraq	ELISA	IgG and IgM	147	136	30 (22.05)	11	5 (45.45)
18	Alawaini *et al*. (2021) [42]	Libya	LAT	IgG	164	73	25 (34.24)	91	30 (32.96)
19	Belkacemi and Heddi (2022) [43]	Algeria	ELISA	IgG and IgM	103	84	38 (45.23)	19	8 (42.1)
20	Fattahi Bafghi *et al*. (2022) [44]	Iran	ELISA	IgG and IgM	368	328	12 (3.65)	40	4 (10)
21	Abdulla *et al*. (2022) [45]	Iraq	ELISA	IgG and IgM	125	113	14 (12.39)	12	2 (16.66)

ELISA: enzyme-linked immunosorbent assay, CFT: complement fixation test, EIA: enzyme immunoassays, LAT: latex agglutination test, IFA: indirect immunofluorescence assay, IgG: immunoglobulin G, IgM: Immunoglobulin M, n: Number, Rh^+^: people with Rh-positive blood group, Rh^-^: people with Rh-negative blood group, Rh^+^ & T^+^: people with Rh-positive blood group and *Toxoplasm*a positive, and Rh^-^ & T^+^: people with Rh-negative blood group and *Toxoplasm*a positive.

### General characteristics of included studies

These cross-sectional studies were conducted in 8 countries [Czech Republic (n = 6), Iraq (n = 5), Iran (n = 4), Egypt (n = 2), and Algeria, Tunisia, Libya, and Ivory Coast (n = 1)], published between 2008 and 2022. *Toxoplasma* antibodies were determined using enzyme-linked immunosorbent assay (ELISA), complement fixation test (CFT), enzyme immunoassays (EIA), latex agglutination test (LAT), and indirect immunofluorescence assay (IFA). A total of 10910 people in Rh-positive and the Rh-negative blood groups were included in the present meta-analysis.

### Quality assessment

The articles that were of moderate [25, 30, 32–36, 38–40, 42, 43, 45] and high quality [19, 22–24, 31, 37, 41, 44] using the NOS checklist were included in the meta-analysis. The quality scores of the various eligible studies are presented in S2 File.

### *Toxoplasma* infection and the Rh-positive blood group

**Fig 2:**
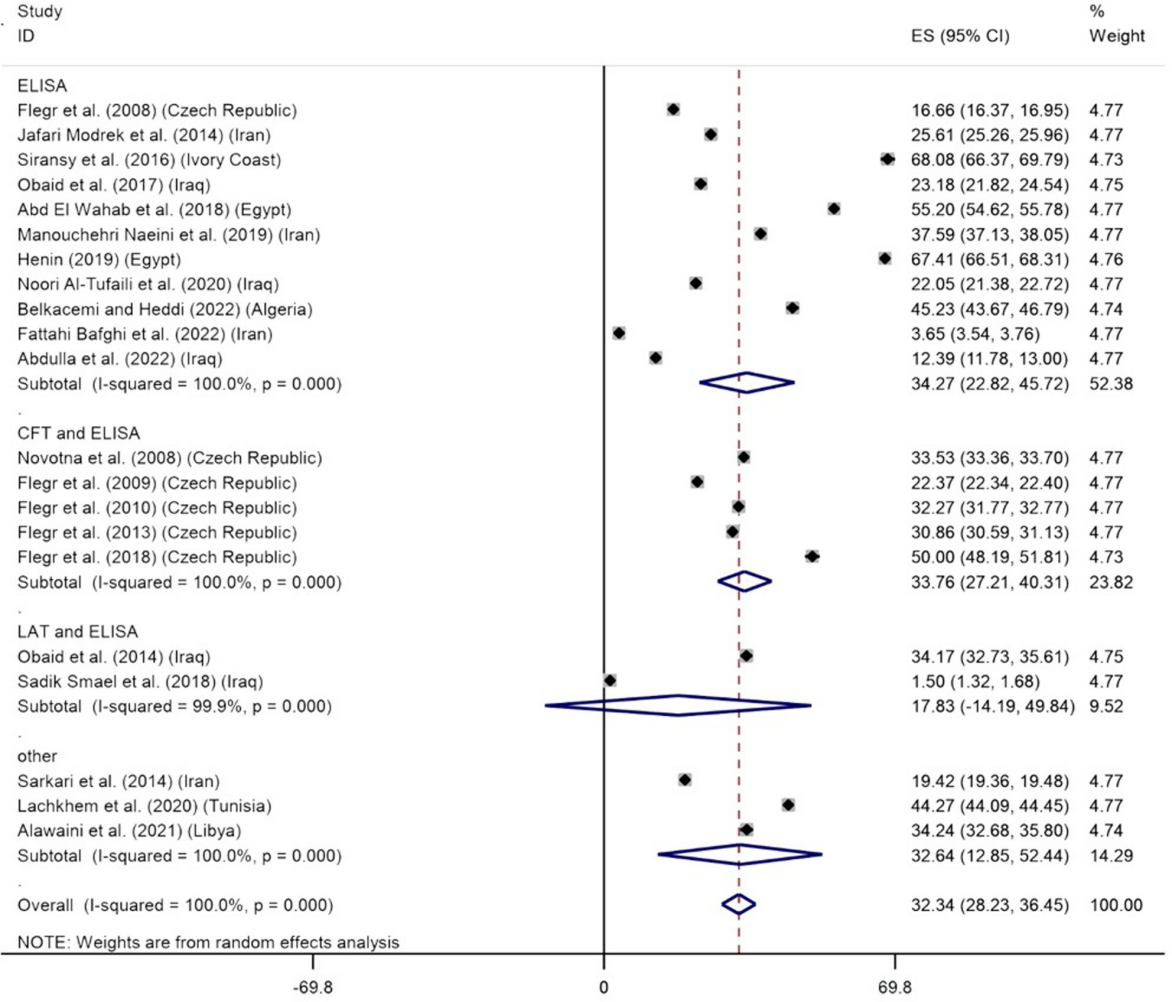
The reported seroprevalence of anti- *T*. *gondii* antibodies in the Rh-positive blood group.

The estimated pooled prevalence of *Toxoplasma* infection in the Rh-positive blood group was 32.34% (CI 95%: 28.23–36.45%). Statistically, significant heterogeneity was observed among studies (I^2^ = 100%, P<0.001) (Fig 2). Egger’s regression test for publication bias showed that it had no significant impact on the estimation of total prevalence (β coefficient: 23.836, P = 0.444) (S1 Fig). A sensitivity analysis was performed by excluding only one study each time to determine whether the change in the inclusion criteria for this meta-analysis had an impact on the results. There was no change in the results (S2 Fig). Based on the meta-analysis, the seroprevalence of *T*. *gondii* infection in the Rh-positive individuals based on the diagnostic methods of ELISA, CFT and ELISA, LAT and ELISA, and others was estimated to be 34.27% (95% CI: 22.82–45.72%), 33.76% (95% CI: 27.21–40.31%), 17.83% (95% CI: -14.19–49.84%), and 32.64% (95% CI: 12.85–52.44%), respectively (Fig 2). These results show that there was a statistically significant difference between the diagnostic methods, except for LAT and ELISA methods, which included only two studies and the difference between them was large. Based on subgroup analysis, no statistically significant difference was observed in the overall prevalence of *T*. *gondii* in Rh-positive individuals based on diagnostic methods (P = 0.488), place of study (P = 0.821), and NOS score (P = 0.858).

### *Toxoplasma* infection and the Rh-negative blood group

**Fig 3:**
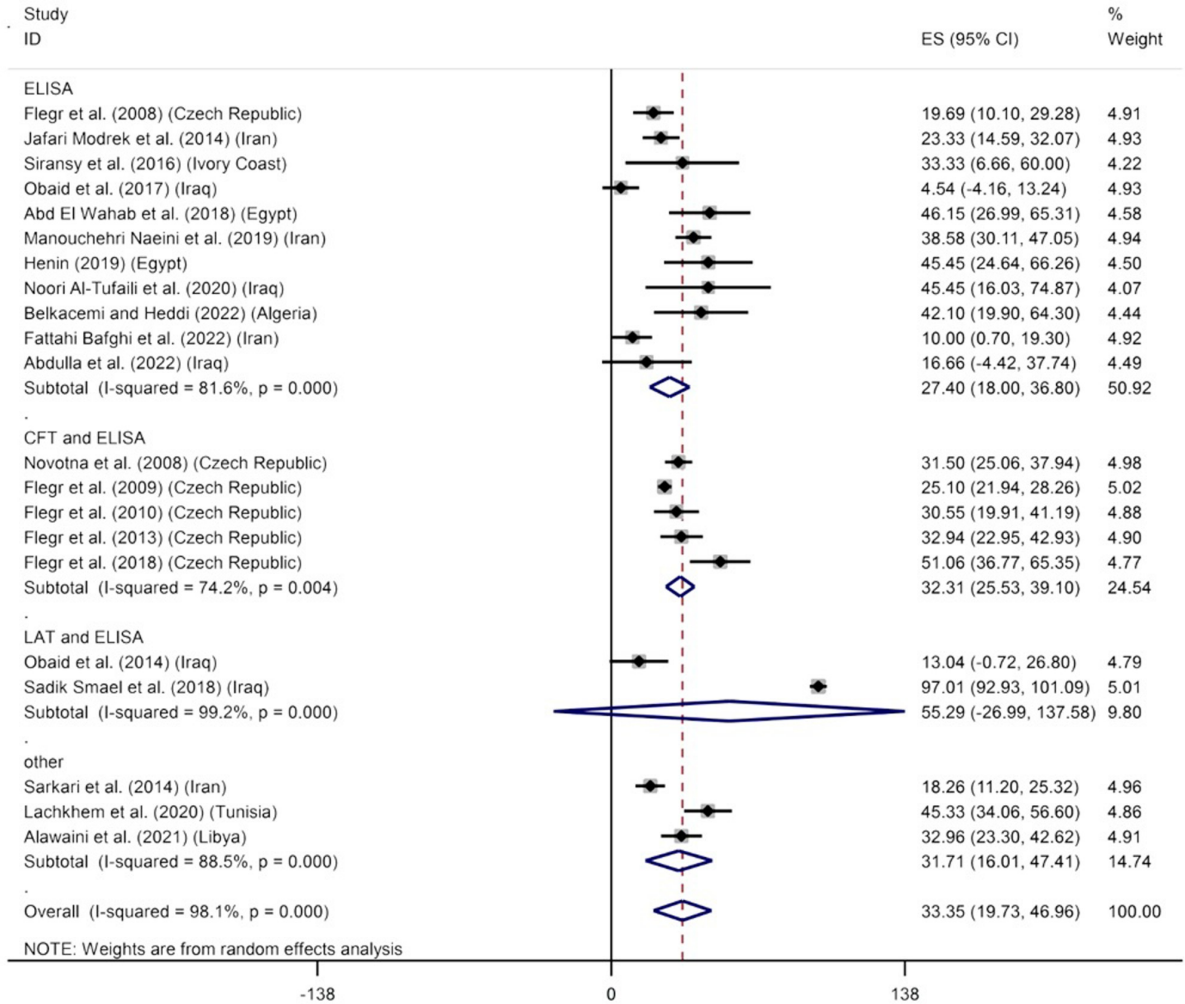
The reported seroprevalence of anti- *T*. *gondii* antibodies in the Rh-negative blood group.

The prevalence of *T*. *gondii* was calculated at 33.35% (CI 95%: 19.73–46.96%) in the Rh-negative blood group. Analysis of heterogeneity found that our meta-analysis had a high heterogeneity (I^2^ = 98.1%, P<0.001) (Fig 3). The results of Egger’s test suggest that there is no significant publication bias (β coefficient: -2.701, P = 0.372) (S3 Fig). Furthermore, removing each study from the meta-analysis through a sensitivity analysis had no impact on the global prevalence of *Toxoplasma* infection in the Rh-negative blood group (S4 Fig). Moreover, the overall prevalence rates of *Toxoplasma* infection based on the diagnostic methods were as follows: ELISA: 27.40% (95% CI: 18.00–36.80%), CFT and ELISA: 32.31% (95% CI: 25.53–39.10%), LAT and ELISA: 55.29% (95% CI: -26.99–137.58%), and other: 31.71% (95% CI: 16.01–47.41%). As shown in Fig 3, there is a statistically significant difference between diagnostic methods except for LAT and ELISA methods. Results of subgroup analysis based on diagnostic methods (P = 0.391), place of study (P = 0.673), and NOS score (P = 0.727) indicated that the effect of these variables on the heterogeneity of studies was not statistically significant.

### Relationship between anti-*T*. *gondii* antibodies and the Rh blood group

**Fig 4:**
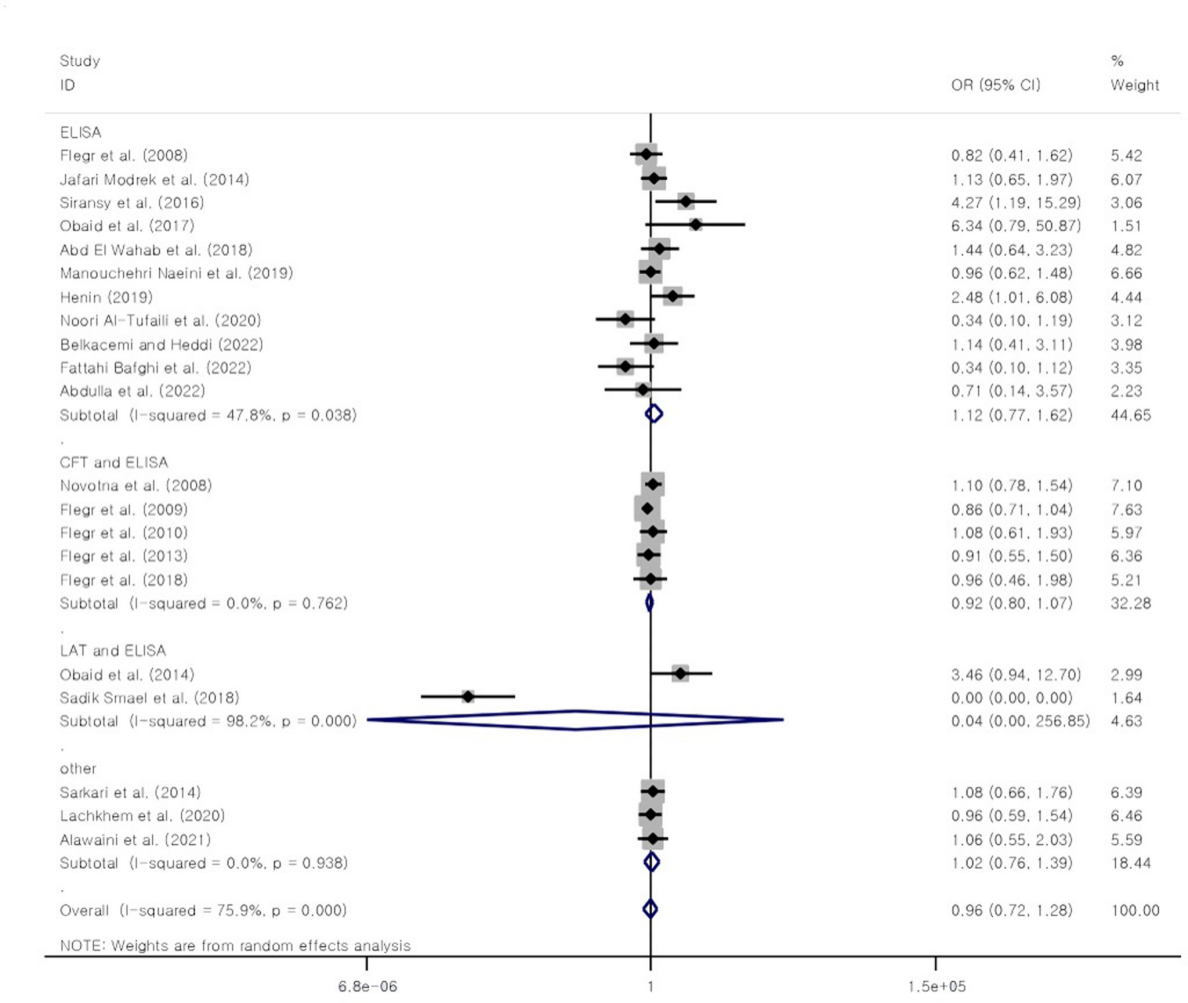
Forest plot of the association between anti-*T*. *gondii* antibodies and the Rh-negative blood group.

The results of this study showed that the overall OR of the relationship between anti-*T*. *gondii* antibodies and the Rh blood group and I^2^ were 0.96 [95% CI: 0.72–1.28] and 75.9%, respectively (Fig 4). Publication bias was assessed with the help of the Egger test (β coefficient: -0.137, P = 0.867) (S5 Fig). The results of the sensitivity analysis showed that removing a study had no impact on the overall prevalence (S6 Fig). Subgroup analysis based on diagnostic methods (P = 0.887), place of study (P = 0.378), and NOS score (P = 0.489) showed that these risk factors do not have a statistically significant effect on the heterogeneity of studies.

## Discussion

In many countries around the world, more than 50% of the population will be infected with *T*. *gondii* during their life [2, 46]. Chronically infected people who carry the protozoan for life differ from healthy people in terms of personality characteristics [47], secondary sex ratio [48], olfactory preferences [49], schizophrenia [50, 51], cancer [52], traffic accidents [53], suicide [11], bipolar disorder [54, 55], obsessive-compulsive disorder [7], Alzheimer’s disease [10, 56], Parkinson’s disease [56], epilepsy [57, 58], autism [9], and headache [8]. The amount of changes usually tends to increase with the duration of toxoplasmosis. Several studies have demonstrated that the severity of toxoplasmosis-related changes is linked to the Rh phenotype of infected patients [22, 23, 25, 26]. Therefore, this study is aimed to determine the seroprevalence of *Toxoplasma* infection in the Rh-positive and the Rh-negative blood groups and evaluate the relationship between the seroprevalence of anti-*Toxoplasma* antibodies and Rh phenotype.

A total of 21 articles on the seroprevalence of *Toxoplasma* infection in Rh blood groups were included in the present meta-analysis. Obtained results indicated the overall seroprevalence of *Toxoplasma* infection is 32.34% and 33.35% in individuals with the Rh-positive and the Rh-negative blood groups.

Considering that positive blood groups (85%) are more common in the human population than negative blood groups (15%), it was thought that the frequency of *Toxoplasma* is higher in the Rh-positive blood group. Nevertheless, the results of this study showed a high seroprevalence of *Toxoplasma* infection in both Rh-negative and positive blood groups. The frequency of Rh-positive phenotypes in Africa and Asia is 95% and 99%, respectively. The highest number of Rh- negative people, about 16%, is in Europe. The frequency of Rh-negative people is different in various parts of the world, for example, it is about 15% in Caucasians, 8% in Africans, and 1% in Asians, which corresponds to the allele frequencies of 40%, 28%, and 10%, respectively [23]. The low abundance of felines and possibly the low prevalence of toxoplasmosis in Europe before the recent advent of the domestic cat could account for the higher prevalence of Rh-negative individuals [23]. In terms of physical health and numerous parameters related to psychomotor and cognitive performance, people without *Toxoplasma* and Rh-negative and even more Rh-positive heterozygotes perform better than Rh-positive homozygotes [22, 59–61]. Therefore, in areas with a low prevalence of toxoplasmosis, such as ancient Europe, the allele for Rh-negative can spread, and on the other hand, in many regions of Africa, Asia, and both continents of the Americas, the prevalence of toxoplasmosis in the human population is much higher due to the abundance of different feline species [62]. Therefore, parasites may play a role in the origin and persistence of the Rh polymorphism by marked differences in the frequency of specific Rh phenotypes in different geographical areas [23].

In the "European" Rh-negative variant, the entire protein-coding part of the gene is deleted [63]. In Rh-negative homozygous cells, no product of this allele is made, and RhCE protein may replace RhD in the relevant molecular complex. As a result, the molecular complexes on the red blood cell membrane and their biological activities are different in Rh-negative and Rh-positive homozygotes. Also, there is an significant difference between the erythrocytes of Rh-positive homozygotes and heterozygotes. The number of D antigen sites on the surfaces of an erythrocyte in Rh homozygotes and heterozygotes is different and is about 33,560 in homozygotes and 17,720 in heterozygotes [64]. RhD-containing and RhD-free complexes may differ in the specificity, activity and most probable response to regulation signals. The membrane pump could directly or indirectly influence the partial tension of oxygen and water balance in different tissues such as the brain [65–67]. Therefore, when faced with different diseases, the sensitivity of Rh-positive homozygotes and Rh-positive heterozygotes can be significantly different. Heterozygotes can be susceptible to one disease and resistant to another, while the opposite may occur in homozygotes. These trade-offs can explain the heterozygous advantage hypothesis [60]. Despite these interpretations, the combined OR of the relationship between anti-*T*. *gondii* antibodies and Rh blood group in this study was 0.96 (95% CI: 0.72–1.28). There was no association between Rh blood groups and seroprevalence of *Toxoplasma* infection. These results show that the Rh-positive blood group in people does not have a protective role against *T*. *gondii*.

The 21 articles included in the present meta-analysis were conducted in three continents of Asia, Europe, and Africa. Considering the location of the articles, six studies were in Europe [Czech Republic], nine were in Asia [Iraq and Iran], and six were in Africa [Egypt, Algeria, Tunisia, Libya, and Ivory Coast]. However, an evident information gap was identified for the continents of America and Australia, and many other countries, where there was no available data on the relationship between *T*. *gondii* and Rh blood groups.

The main limitation of the studies included in this meta-analysis is that participants were only screened for the Rh phenotype and not the Rh genotype. Novotná *et al*. showed that Rh-positive heterozygotes are resistant to the physiological effects of toxoplasmosis, and Rh-positive homozygotes are only transiently protected against some of the negative effects of toxoplasmosis [22]. More informative data will probably be obtained when Rh-positive participants are tested for their Rh genotype. Determining the Rh phenotype is very easy and inexpensive using the standard agglutination technique. However, a much more sophisticated and expensive technique must be used to determine the Rh genotype [30]. Some studies have shown that the Rh-*Toxoplasma* interaction in men, despite a relatively large effect size, lacks a significant effect. Considering that the number of Rh-negative and *Toxoplasma*-positive male participants, which are the rarest subpopulation in the general population, is relatively small [62].

The lack of information about the Rh genotype of individuals makes any speculation about the mechanism of Rh positivity protection extremely difficult. Regarding the localization and possible function of RH proteins, previous studies [22, 23, 68] reported that RhD and RhCE proteins are encoded in the RH locus and are located on the red blood cell membrane (as ion pumps) and are involved in the regulation of ion balance in some vital parts of nerve or muscle tissue. These regulations are important in the presence of *Toxoplasma* cysts in the nerve and muscle tissues of subjects, especially disabled people [20, 21, 25].

In addition, high heterogeneity was reported in this systematic review and meta-analysis. A source of heterogeneity is the difference in the genetic potential of the target population, which is influenced by lifestyle and environmental factors such as dietary habits, environmental pollution, exposure to ultraviolet radiation, types of infections, and socioeconomic factors. The age of the study participants is another source of heterogeneity. Because old age increases the possibility of exposure to *Toxoplasma*. The variety of diagnostic methods may also be a source of heterogeneity. In our study, the results of the meta-regression test showed that the diagnostic methods do not have a significant effect on the heterogeneity of the studies. The limitations of this study are 1) the small sample size in the included studies, 2) the use of English articles due to lack of fluency in other languages, 3) the presence of heterogeneity, 4) the diversity of the diagnostic tests with different sensitivity and specificity, 5) data analysis using phenotype and not genotype, and 6) the lack of the evaluation of risk factors, such as age, gender, race, etc.

The results of this study showed that there was no statistically significant difference between the prevalence of *Toxoplasma* infection in Rh-positive and Rh-negative people and the prevalence of infection was high in both blood groups. Also, there was no relationship between Rh blood groups and the seroprevalence of *Toxoplasma* infection, which indicates the lack of protective role of the Rh-positive blood group in people against *T*. *gondii*. Considering the small number of studies that directly examined the relationship between toxoplasmosis and Rh factor, more and better quality studies in the future to determine this relationship may lead to better insight to clarify the possible relationship between toxoplasmosis and Rh blood group.

## Supporting information

S1 Checklist PRISMA 2009 checklist. (DOC)

S1 File Search strategy. (DOCX)

S2 File NOS checklist. (DOCX)

S3 File (DOCX)

S1 Fig Funnel plot to detect publication bias in studies showing seroprevalence of *Toxoplasma* infection in the Rh-positive blood group. (TIF)

S2 Fig Sensitivity analysis for assessing the effect of each primary study on the total estimates in studies showing seroprevalence of *Toxoplasma* infection in the Rh-positive blood group. (TIF)

S3 Fig Funnel plot to detect publication bias in studies showing seroprevalence of *Toxoplasma* infection in the Rh-negative blood group. (TIF)

S4 Fig Sensitivity analysis for assessing the effect of each primary study on the total estimates in studies showing seroprevalence of *Toxoplasma* infection in the Rh-negative blood group. (TIF)

S5 Fig Funnel plot to detect publication bias in studies showing the association between anti-*T*. *gondii* antibodies and the Rh blood group. (TIF)

S6 Fig Sensitivity analysis for assessing the effect of each primary study on the association between anti-*T*. *gondii* antibodies and the Rh blood group. (TIF)
