# Supplementary material for: Toxoplasma infection and Rhesus blood group system: A systematic review and meta-analysis
Source: PLoS One. 2023 Jul 5;18(7):e0287992. doi: 10.1371/journal.pone.0287992 (PMC10321609; doi:10.1371/journal.pone.0287992)
Supplement: S1 File — (DOCX) [file pone.0287992.s002.docx]

Supplementary Table 1. Search strategy in PubMed.

| Search | Search terms and combinations |
| --- | --- |
| 1 | (“Toxoplasma gondii” OR toxoplasmosis) |
| 2 | ("blood donors" OR "blood group" OR Rhesus OR RhD) |
| 4 | #1 AND #2 |
| 5 | Filters: Full text, Journal article, English language |

Supplementary Table 2. Search strategy in ScienceDirect.

| Search | Search terms and combinations |
| --- | --- |
| 1 | (“Toxoplasma gondii” OR toxoplasmosis) |
| 2 | ("blood donors" OR "blood group" OR Rhesus OR RhD) |
| 4 | #1 AND #2 |
| 5 | Filters: Research articles |

Supplementary Table 3. Search strategy in ProQuest.

| Search | Search terms and combinations |
| --- | --- |
| 1 | (“Toxoplasma gondii” OR toxoplasmosis) |
| 2 | ("blood donors" OR "blood group" OR Rhesus OR RhD) |
| 4 | #1 AND #2 |
| 5 | Filters: Full text, Publication data: All data, Source type: Scholarly Journals, Document type: Article, Language: English language |

Supplementary Table 4. Search strategy in Google Scholar

| Search | Search terms and combinations |
| --- | --- |
| 1 | (“Toxoplasma gondii” OR toxoplasmosis) |
| 2 | ("blood donors" OR "blood group" OR Rhesus OR RhD) |
| 4 | #1 AND #2 |
| 5 | Filters: Language: English language |
