# Supplementary material for: Toxoplasma infection and Rhesus blood group system: A systematic review and meta-analysis
Source: PLoS One. 2023 Jul 5;18(7):e0287992. doi: 10.1371/journal.pone.0287992 (PMC10321609; doi:10.1371/journal.pone.0287992)
Supplement: S2 File — (DOCX) [file pone.0287992.s003.docx]

**Table S2.** Quality assessment of included cross-sectional studies based on the Newcastle-Ottawa Scale.

| Id | First author | Publication year | Place of study | Selection (3 points) | Comparability (2 points) | Outcome (2 points) | Total (scores) | Quality category |
| --- | --- | --- | --- | --- | --- | --- | --- | --- |
| 1 | Flegr *et al.* | 2008 | Czech Republic | 3 | 2 | 2 | 7 | High quality |
| 2 | Novotna *et al.* | 2008 | Czech Republic | 3 | 2 | 2 | 7 | High quality |
| 3 | Flegr *et al.* | 2009 | Czech Republic | 2 | 1 | 2 | 5 | Moderate quality |
| 4 | Flegr *et al.* | 2010 | Czech Republic | 3 | 2 | 2 | 7 | High quality |
| 5 | Flegr *et al.* | 2013 | Czech Republic | 2 | 0 | 2 | 4 | Moderate quality |
| 6 | Obaid *et al.* | 2014 | Iraq | 2 | 2 | 2 | 6 | High quality |
| 7 | Sarkari *et al.* | 2014 | Iran | 3 | 0 | 2 | 5 | Moderate quality |
| 8 | Jafari Modrek *et al.* | 2014 | Iran | 3 | 0 | 2 | 5 | Moderate quality |
| 9 | Siransy *et al.* | 2016 | Ivory Coast | 3 | 0 | 2 | 5 | Moderate quality |
| 10 | Obaid *et al.* | 2017 | Iraq | 3 | 0 | 2 | 5 | Moderate quality |
| 11 | Abd El Wahab *et al.* | 2018 | Egypt | 3 | 0 | 2 | 5 | Moderate quality |
| 12 | Sadik Smael *et al.* | 2018 | Iraq | 2 | 2 | 2 | 6 | High quality |
| 13 | Flegr *et al.* | 2018 | Czech Republic | 2 | 2 | 2 | 6 | High quality |
| 14 | Manouchehri Naeini *et al.* | 2019 | Iran | 3 | 0 | 2 | 5 | Moderate quality |
| 15 | Henin | 2019 | Egypt | 3 | 0 | 2 | 5 | Moderate quality |
| 16 | Lachkhem *et al.* | 2020 | Tunisia | 3 | 0 | 2 | 5 | Moderate quality |
| 17 | Noori Al-Tufaili *et al.* | 2020 | Iraq | 2 | 2 | 2 | 6 | High quality |
| 18 | Alawaini *et al.* | 2021 | Libya | 3 | 0 | 2 | 5 | Moderate quality |
| 19 | Belkacemi and Heddi | 2022 | Algeria | 3 | 0 | 2 | 5 | Moderate quality |
| 20 | Fattahi Bafghi *et al.* | 2022 | Iran | 2 | 2 | 2 | 6 | High quality |
| 21 | Abdulla *et al.* | 2022 | Iraq | 2 | 1 | 2 | 5 | Moderate quality |
