# Supplementary material for: Toxoplasma infection and Rhesus blood group system: A systematic review and meta-analysis
Source: PLoS One. 2023 Jul 5;18(7):e0287992. doi: 10.1371/journal.pone.0287992 (PMC10321609; doi:10.1371/journal.pone.0287992)
Supplement: S3 File — (DOCX) [file pone.0287992.s004.docx]

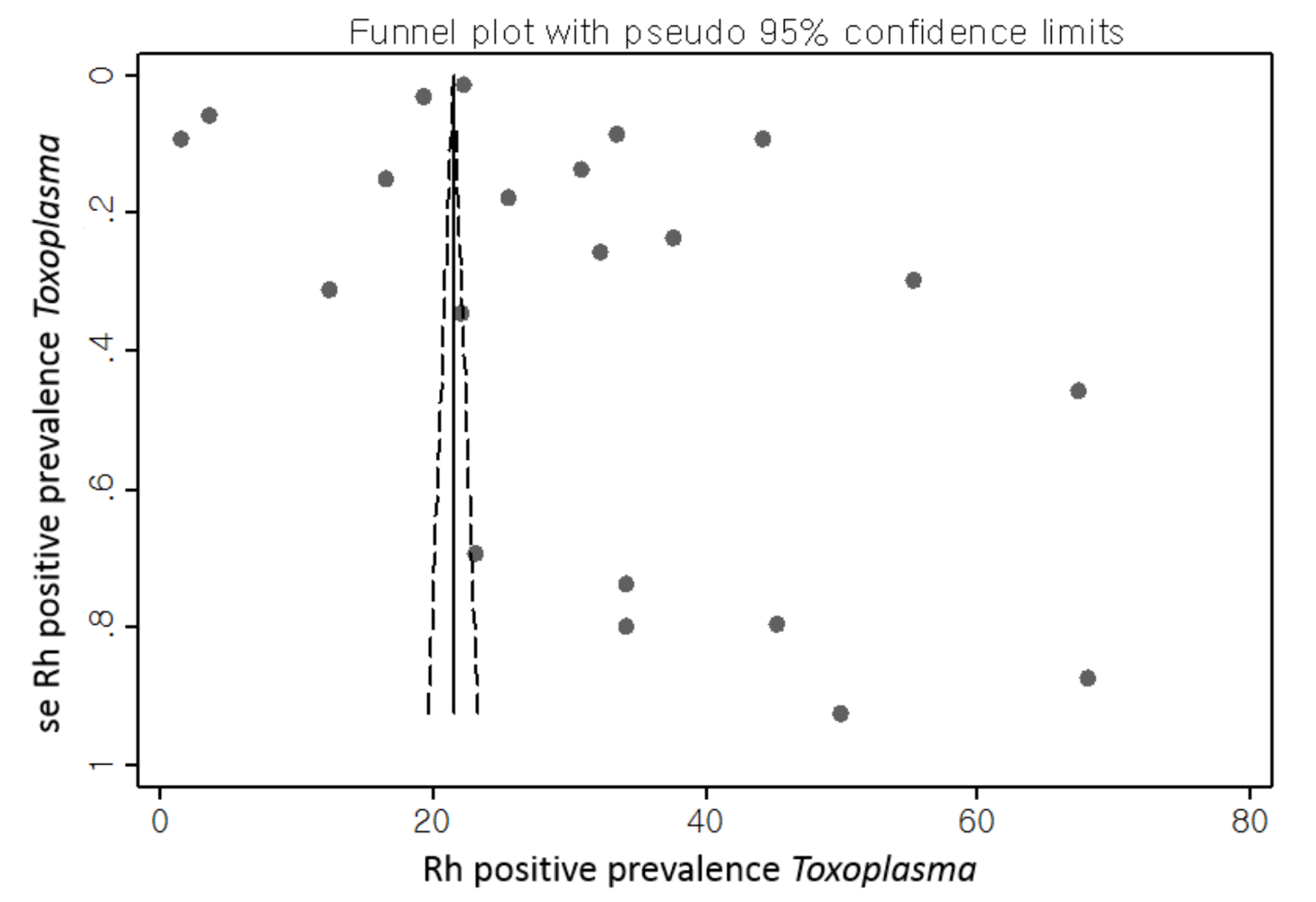


**Supplementary Fig 1.** Funnel plot to detect publication bias in studies showing seroprevalence of *Toxoplasma* infection in the Rh-positive blood group. (s.e.: standard error)

**Supplementary Fig 2.** Sensitivity analysis for assessing the effect of each primary study on the total estimates in studies showing seroprevalence of *Toxoplasma* infection in the Rh-positive blood group.

**
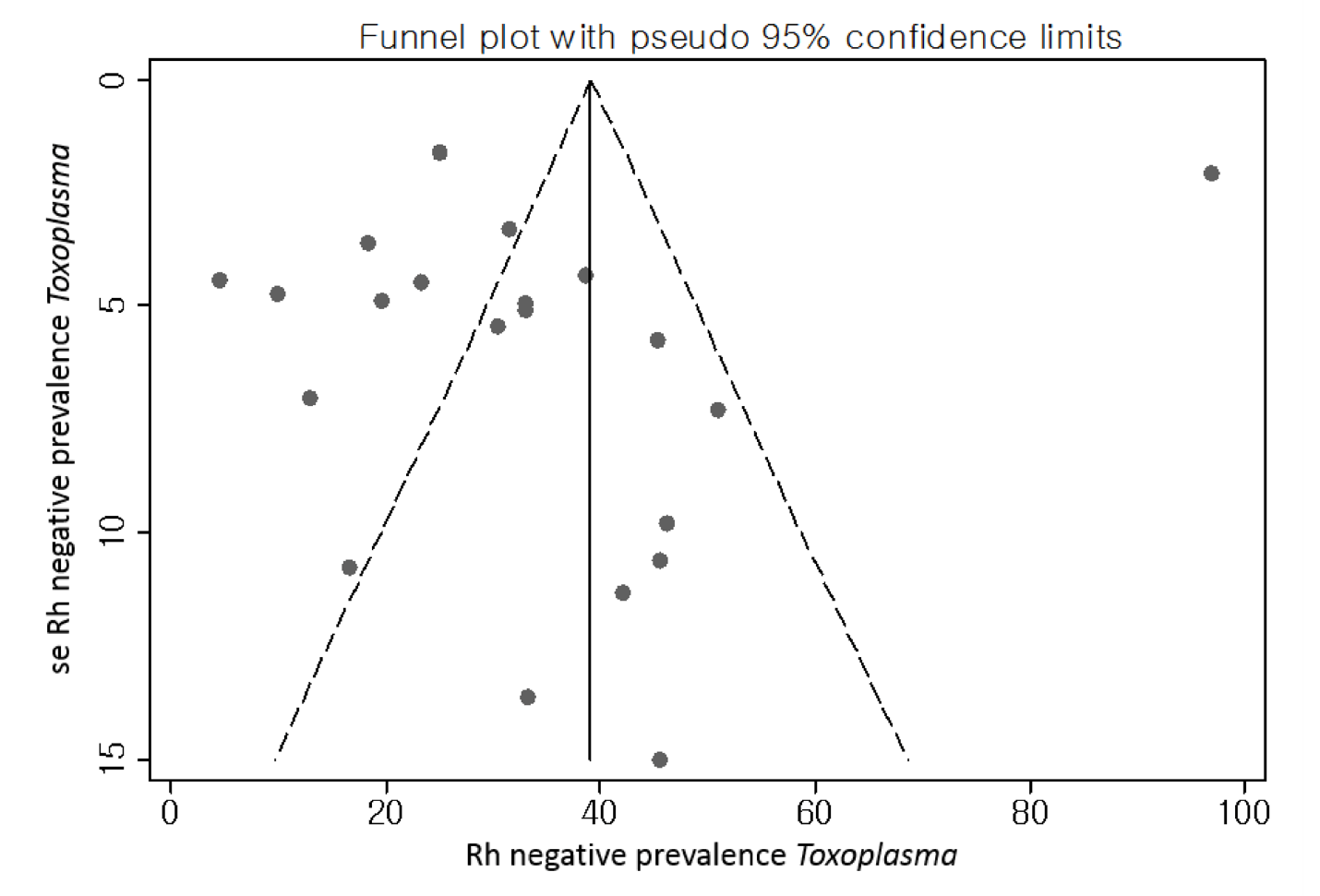
**

**Supplementary Fig 3.** Funnel plot to detect publication bias in studies showing seroprevalence of *Toxoplasma* infection in the Rh-negative blood group. (s.e.: standard error)

**
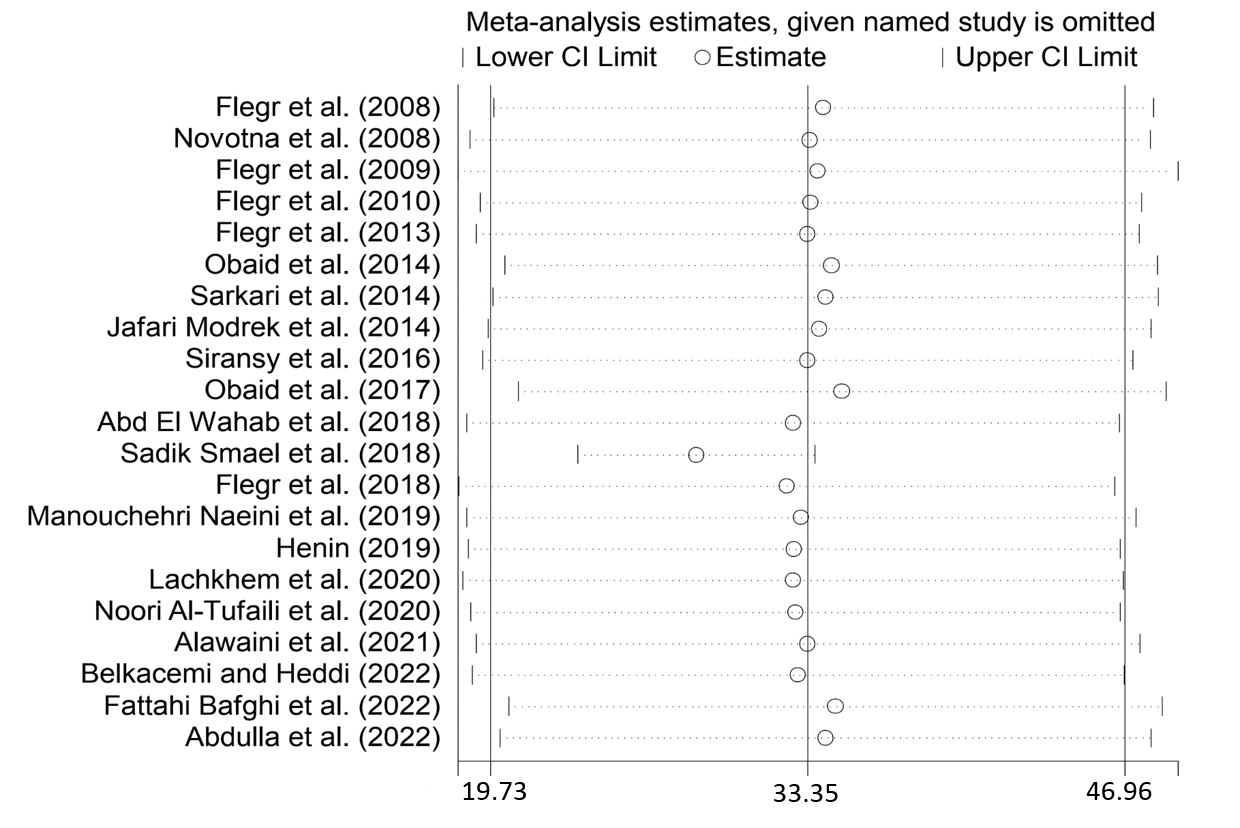
**

**Supplementary Fig 4.** Sensitivity analysis for assessing the effect of each primary study on the total estimates in studies showing seroprevalence of *Toxoplasma* infection in the Rh-negative blood group.

**Supplementary Fig 5.** Funnel plot to detect publication bias in studies showing the association between anti-*T. gondii* antibodies and the Rh blood group. (s.e.: the standard error and OR: odds ratio)

**Supplementary Fig 6.** Sensitivity analysis for assessing the effect of each primary study on the association between anti-*T. gondii* antibodies and the Rh blood group.
