# Supplementary figures and images for: Toxoplasma infection and Rhesus blood group system: A systematic review and meta-analysis
Source: PLoS One. 2023 Jul 5;18(7):e0287992. doi: 10.1371/journal.pone.0287992 (PMC10321609; doi:10.1371/journal.pone.0287992)

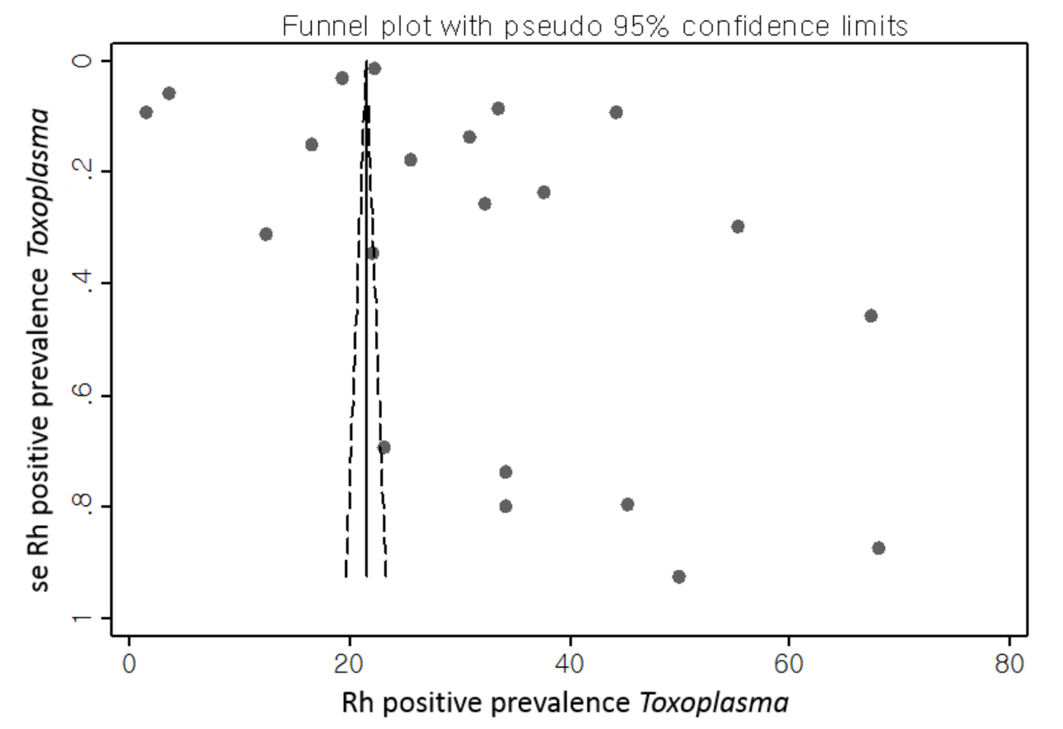

Supplement: S1 Fig — (TIF) [file pone.0287992.s005.tif]

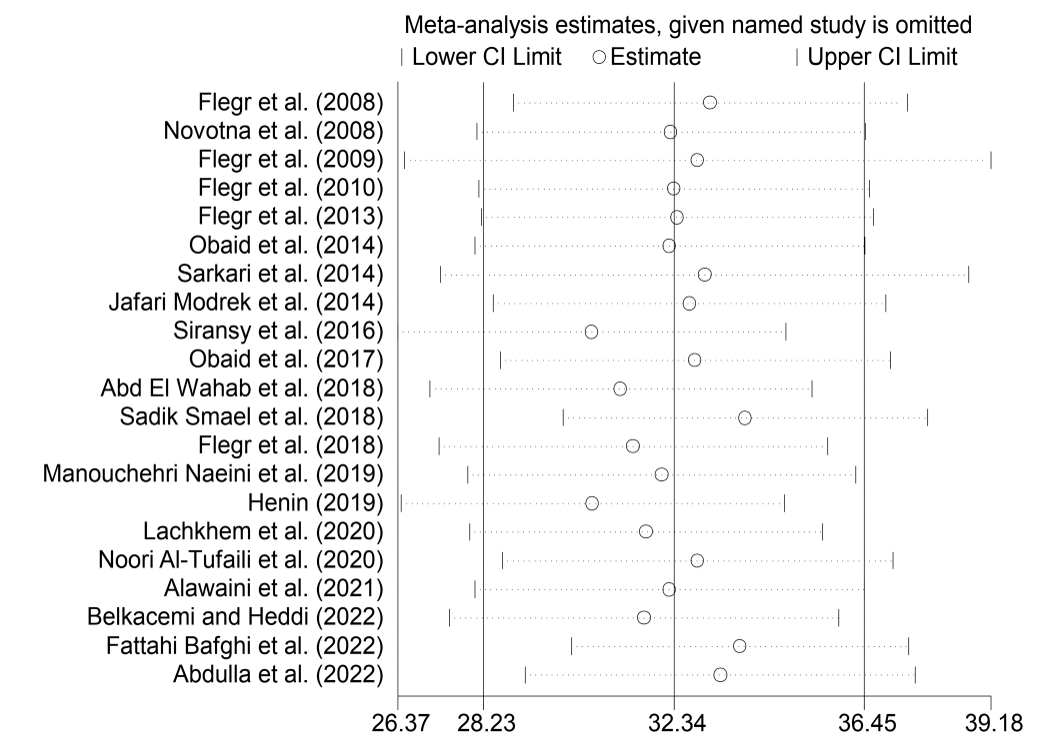

Supplement: S2 Fig — (TIF) [file pone.0287992.s006.tif]

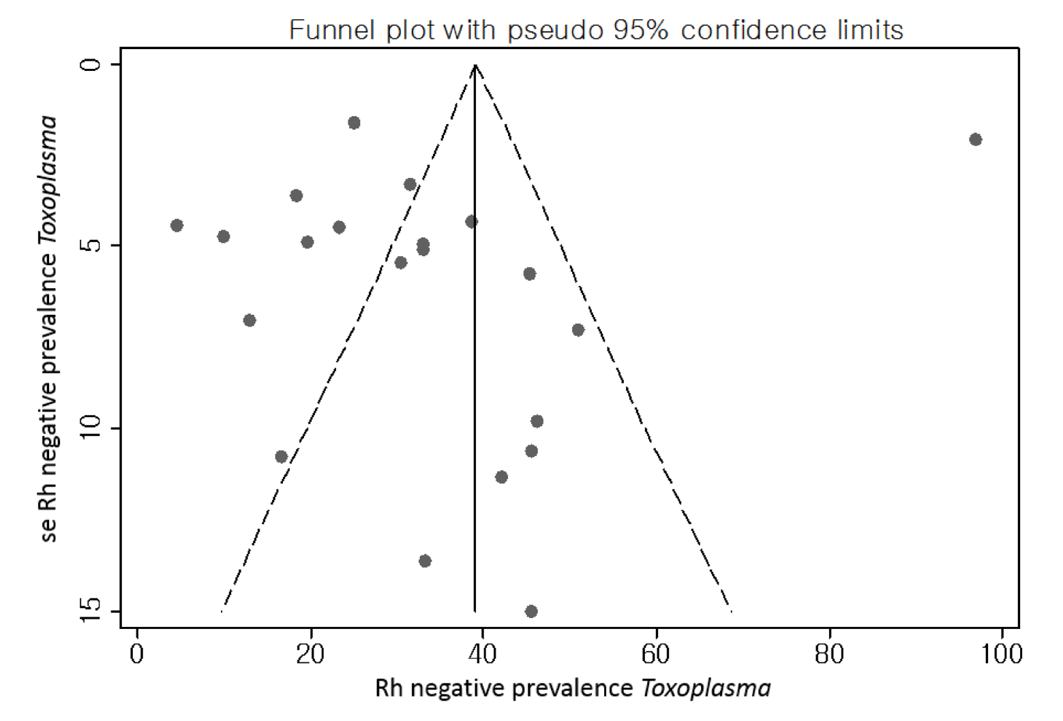

Supplement: S3 Fig — (TIF) [file pone.0287992.s007.tif]

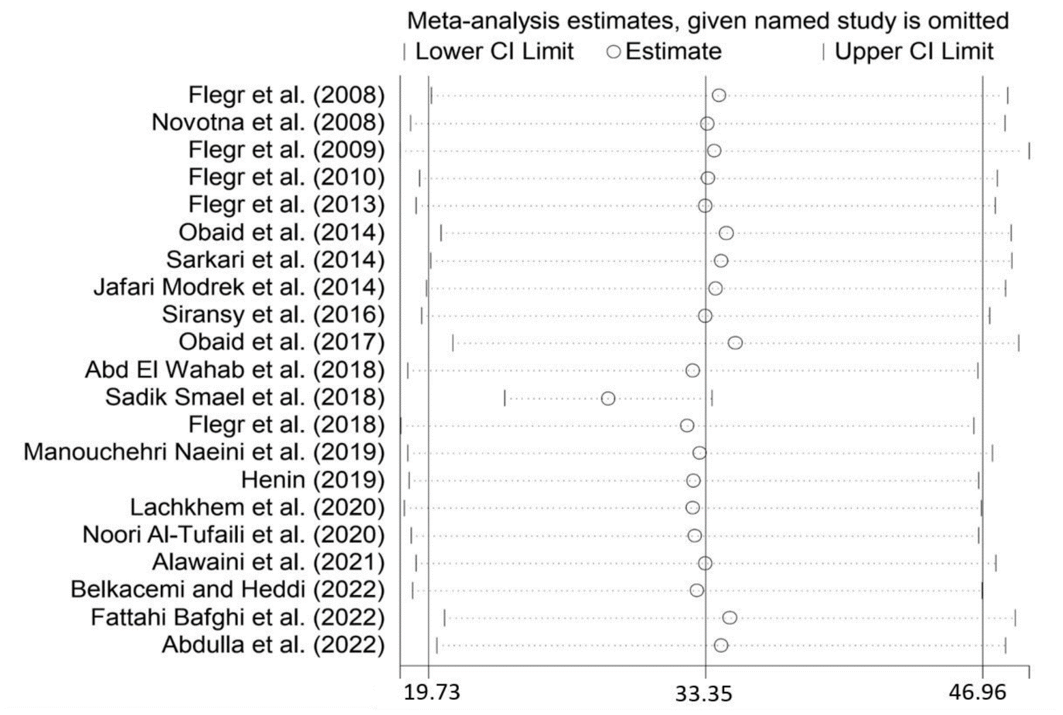

Supplement: S4 Fig — (TIF) [file pone.0287992.s008.tif]

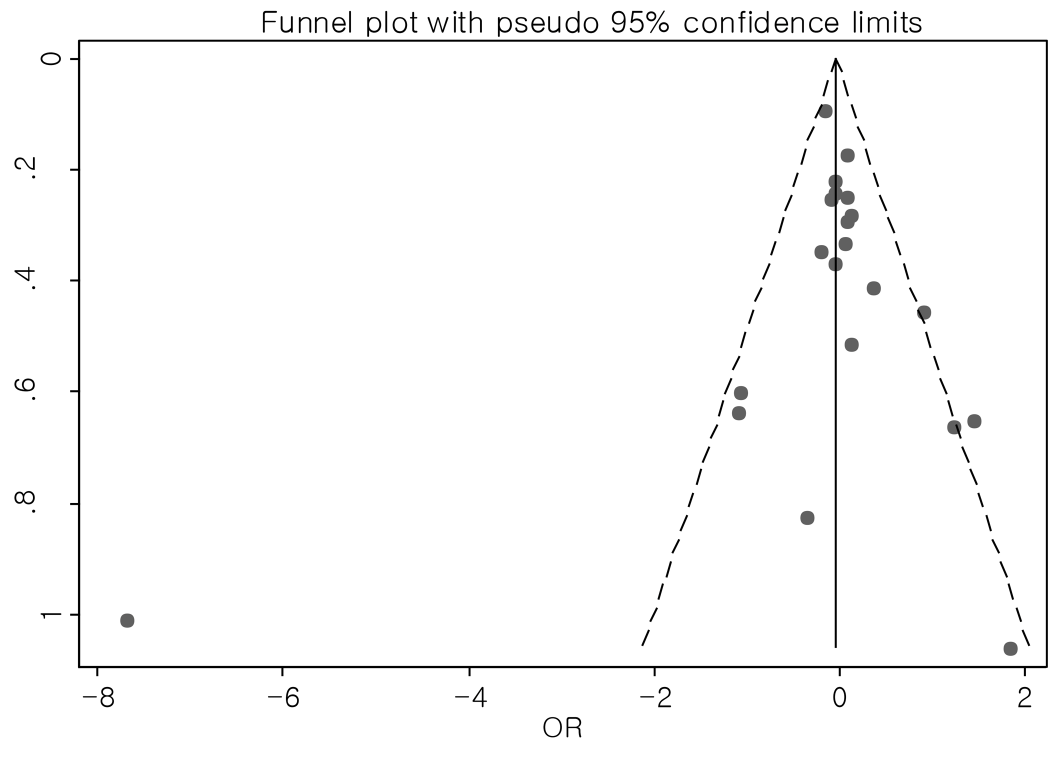

Supplement: S5 Fig — (TIF) [file pone.0287992.s009.tif]

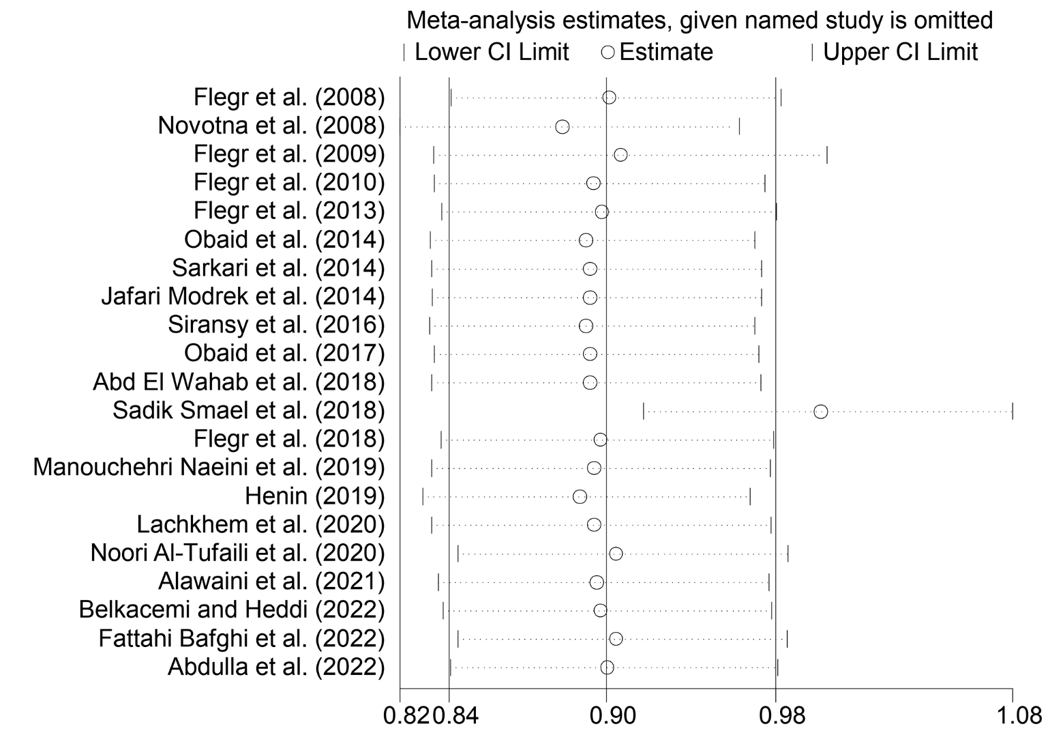

Supplement: S6 Fig — (TIF) [file pone.0287992.s010.tif]
